# HemeBIND: a novel method for heme binding residue prediction by combining structural and sequence information

**DOI:** 10.1186/1471-2105-12-207

**Published:** 2011-05-26

**Authors:** Rong Liu, Jianjun Hu

**Affiliations:** 1 Department of Computer Science and Engineering, University of South Carolina, Columbia, SC 29208, USA

## Abstract

**Background:**

Accurate prediction of binding residues involved in the interactions between proteins and small ligands is one of the major challenges in structural bioinformatics. Heme is an essential and commonly used ligand that plays critical roles in electron transfer, catalysis, signal transduction and gene expression. Although much effort has been devoted to the development of various generic algorithms for ligand binding site prediction over the last decade, no algorithm has been specifically designed to complement experimental techniques for identification of heme binding residues. Consequently, an urgent need is to develop a computational method for recognizing these important residues.

**Results:**

Here we introduced an efficient algorithm HemeBIND for predicting heme binding residues by integrating structural and sequence information. We systematically investigated the characteristics of binding interfaces based on a non-redundant dataset of heme-protein complexes. It was found that several sequence and structural attributes such as evolutionary conservation, solvent accessibility, depth and protrusion clearly illustrate the differences between heme binding and non-binding residues. These features can then be separately used or combined to build the structure-based classifiers using support vector machine (SVM). The results showed that the information contained in these features is largely complementary and their combination achieved the best performance. To further improve the performance, an attempt has been made to develop a post-processing procedure to reduce the number of false positives. In addition, we built a sequence-based classifier based on SVM and sequence profile as an alternative when only sequence information can be used. Finally, we employed a voting method to combine the outputs of structure-based and sequence-based classifiers, which demonstrated remarkably better performance than the individual classifier alone.

**Conclusions:**

HemeBIND is the first specialized algorithm used to predict binding residues in protein structures for heme ligands. Extensive experiments indicated that both the structure-based and sequence-based methods have effectively identified heme binding residues while the complementary relationship between them can result in a significant improvement in prediction performance. The value of our method is highlighted through the development of HemeBIND web server that is freely accessible at http://mleg.cse.sc.edu/hemeBIND/.

## Background

The heme cofactor, an extremely versatile prosthetic group, is essential and important for virtually all organisms [1]. Hemes can be classified into different types based on their chemical structures. In nature, the most common type is *b*-type and its derivatives such as *a*-, *c*-, *d*-, and *o*-type, all use *b*-type as a template [2]. Heme cofactors are usually bound by heme proteins, which play an important role in a wide variety of biological processes, including electron transfer [3], oxygen transport [4], metal ion storage [5], chemical catalysis [6], gene expression [7], and cellular signaling [8]. Identification of residues involved in heme binding sites can help to better understand the biological functions of heme proteins, to uncover the mechanism of heme-protein interactions, and to provide valuable clues for bio-inspired protein design. However, experimental determination of heme binding residues is time-consuming and labor-intensive. It is therefore highly desirable to develop computational methods capable of predicting these important residues.

Over the past fifteen years, a large number of computational approaches have been developed to analyze and predict small ligand binding sites. Broadly, from the perspective of feature extraction, these methods can be divided into three categories: structure-based methods, sequence-based methods, and hybrid methods that combine both structural and sequence information. Among structure-based methods, geometric approaches are widely proposed to detect protein binding pockets, including POCKET [9], LIGSITE [10], SURFNET [11], CAST [12], and PocketPicker [13]. These algorithms extract solvent accessible pockets on the protein surface and rank them by some geometric measures such as volume, for arranging top-ranked pockets as the putative binding sites. Alternatively, energy-based methods are also commonly used in identifying ligand binding sites when structural information is available. Q-SiteFinder [14] is an excellent example, which adds hydrophobic (CH3) probes to the protein for calculating van der Waals interaction energy and considers the clusters of probes with the most favorable interaction energy as the potential binding sites. On the other hand, sequence-based approaches such as Rate4Site [15] and ConSurf [16] have largely exploited evolutionary conservation of binding site motifs, or the tendency of functionally important residues to accept fewer mutations compared with the rest of the protein. Recently, more and more methods attempted to recognize ligand binding sites by integrating both structural and sequence information. For example, LIGSITE^CSC ^[17], SURFNET-ConSurf [18], and ConCavity [19] all incorporated residue evolutionary conservation into pocket detection. Additionally, FINDSITE [20] used protein threading to evaluate binding site conservation across groups of weakly homologous template structures. Subsequently, NCBI IBIS sever [21] was built to cluster binding sites found in homologous proteins based on their sequence and structure conservation to annotate different types of binding partners for a query protein. In summary, these computational approaches have achieved success at different levels in ligand binding site prediction.

However, most of the aforementioned methods focused on predicting general ligand binding sites without considering the differences in various ligands. In fact, protein binding sites vary in their roles in different types of protein-ligand interactions [22]. Accordingly, separate consideration should be given for specialized ligand types. Several research groups have developed such ligand-specific binding site prediction algorithms. Sodhi et al. [23] presented a neural network based algorithm to predict the binding residues of six common metal ions using position specific scoring matrix (PSSM), secondary structure, solvent accessibility, and the inter-atomic distance matrix. Guo et al. [24] applied support vector machine (SVM) combined with a novel statistical descriptor (the Oriented Shell Model) containing various physicochemical properties to identify ATP-binding sites. Nebel et al. [25] reported a method to automatically generate structural motifs of protein binding sites on the basis of consensus atom positions and evaluated it on adenine-based ligands. Bordner [26] developed a group of random forest classifiers to predict the binding sites in protein structures for specific metal ions or small molecules using diverse residue-based properties. In addition, Raghava's group constructed four web servers based on SVM and PSSM to predict the binding residues of ATP, GTP, FAD and NAD ligands respectively only using protein sequence information [27-30]. Nevertheless, to our knowledge, no computational method has been developed for specifically detecting the binding residues interacting with heme ligands.

In this paper, a novel algorithm HemeBIND is proposed for identification of heme binding residues by combining structural and sequence information. First, we provided a detailed analysis of various properties of heme binding residues compared with other residues of the protein, such as interface propensity, evolutionary conservation, solvent accessibility, depth, protrusion and spatial clustering of binding residues, based on a non-redundant dataset of *b*- and *c*-type heme proteins. It was found that these features have distinctly different distributions between heme binding and non-binding residues. We then constructed and evaluated a set of structure-based classifiers by using sequence profile, solvent accessibility, depth, protrusion or the combinations of them as the input features of SVMs for heme binding residue prediction. The results showed that these four features provide largely complementary information and their combination achieved the best prediction performance. To further improve the performance, a post-processing procedure was developed to reduce false positives generated by the structure-based classifier with the combined four features. Next, we constructed a sequence-based classifier based on SVM and sequence profile as an alternative method, which is useful when only sequence information is available. Finally, a simple ensemble algorithm was proposed by combining the predictions of the structure-based and sequence-based classifiers, yielding a substantial improvement in prediction performance. Extensive experiments demonstrated that the proposed method can be successfully applied to the prediction of heme binding residues and could provide valuable insights into binding residue prediction for other types of ligands.

## Methods

### Dataset preparation

#### Main dataset

To construct the dataset of heme proteins, we extracted 2209 heme-protein complexes, mainly composed of *b*- and *c*-type hemes, by using "HEM" as a HET group code to search against the Het-PDB Navi. Database (version at May 2010) [31]. Only the X-ray diffraction protein structures with a resolution better than 3Å were reserved in the current study. In order to reduce sequence redundancy, 4127 heme proteins from the selected complexes were compared using the BLASTCLUST program [32]. Two chains were assigned to the same cluster if the sequence identity was more than 30% and the alignment length covered at least 90% of one member of a chain pair. As a result, these heme proteins were classified into 147 clusters. For each cluster, we chose the longest heme protein as a representative. Because five heme proteins (155C:A, 2OLP:A, 3CAO:A, 4CAT:A, 4CAT:B) contain "X" amino acid and the structural file of one heme protein (1C53:A) can not be calculated by the DSSP program [33], these chains were excluded. Therefore, the main dataset is composed of 141 non-redundant heme proteins (Additional file 1, Table S1).

#### Alternative dataset

In addition to the main dataset, we constructed an alternative dataset derived from the experimental data prepared by Fufezan et al. [2]. The original dataset consists of 89 heme proteins, where no two chains have more than 25% sequence identity. We found that the HET group codes of 14 records are not labeled as "HEM". To keep consistent with the main dataset, these chains have been removed from the original dataset. Thus, the remaining 75 heme proteins were used as our alternative dataset (Additional file 1, Table S2).

#### Independent test set

Since the heme proteins in the study of Fufezan et al. [2] were collected in March 2007, the chains collected afterwards in our main dataset can be considered as an independent test set to evaluate our method by using the alternative dataset as a training set. Hence, from the main dataset, the chains sharing more than 30% sequence identity with any one of the 75 chains in the alternative dataset were eliminated. As a result, we obtained a non-redundant set of 72 heme proteins. In this dataset, 62 protein chains bind a single heme molecule and 10 protein chains interact with multiple heme molecules, respectively (Additional file 1, Table S3).

### Extraction of heme binding residues

In this study, following the step of Raghava et al.'s work [27-30], we used the Ligand Protein Contact (LPC) server [34] to arrange heme binding and non-binding residues for the protein chains in our three datasets. The LPC server utilizes the surface complementarity approach developed by Sobolev et al. [35] to define the contacts in protein-ligand complexes. For the protein chains binding multiple heme molecules, we considered all the residues forming contacts with these ligands as the binding residues in the given chain. According to the analysis of LPC server, the main dataset contains 5079 binding residues and 32712 non-binding residues, the alternative dataset includes 2512 binding residues and 16045 non-binding residues, and the independent test set has 2652 binding residues and 15904 non-binding residues, respectively. It should be emphasized that since our prediction method attempts to take advantage of structural information, the residues that have no atomic coordinates were not used in the present work.

### Feature generation

#### Position specific scoring matrix (PSSM)

PSSM is commonly used to measure residue evolutionary conservation in a particular protein of interest. The elements in this matrix represent the probability of 20 residue types occurring at each position in the multiple sequence alignment of the given protein and its homologs. In our work, the PSSM of each heme protein was generated by three iterations of PSI-BLAST [32] searches against NCBI non-redundant database with the BLOSUM62 substitution matrix and E-value threshold of 0.001. The elements of PSSM were scaled between 0 and 1 by the standard logistic function [36]:(1)

where x is the raw matrix value.

#### Relative accessible surface area (RASA)

The solvent accessible surface area (SASA) is the atomic surface area of a molecule that is in contact with solvent. Herein the SASA of each residue in heme proteins was calculated using the DSSP program [33]. It should be noted that only the atomic coordinates of the unbound chain were extracted for the calculation. To obtain the RASA of each residue, the absolute values were scaled between 0 and 1 by the following equation [37]:(2)

where SASA_r _is the SASA of residue r, max(SASA_r_) is the maximum SASA of residue r defined by Rost and Sander [38].

#### Depth index (DPX)

DPX is defined as the distance between a given atom and its closest solvent accessible atom (SASA > 0). Hence, the depth is zero for solvent accessible atoms and greater than zero for interior atoms, and deeply buried atoms have higher DPX values [39]. In our study, the PSAIA software [40] with default parameters was utilized to generate the DPX-related features of each residue in the unbound chain that include the average of all atom DPXs, the standard deviation of all atom DPXs, the average of all side-chain atom DPXs, the standard deviation of all side-chain atom DPXs, the minimal atom DPX and maximal atom DPX. These features were scaled between 0 and 1 using the standard logistic function.

#### Protrusion index (CX)

CX is another important measure used to describe the geometric shape of a protein, reflecting the extent to which an atom protrudes from the protein surface [41]. For each heavy atom in a protein structure, a sphere of predetermined radius is centered around it, and the ratio (CX) between the volume occupied by the protein and the remaining volume within the sphere is calculated. The PSAIA software re-implemented the CX algorithm developed by Pintar et al. [42]. Thus, the CX-related features of each residue, including the average of all atom CXs, the standard deviation of all atom CXs, the average of all side-chain atom CXs, the standard deviation of all side-chain atom CXs, the minimal atom CX and maximal atom CX, were calculated using this software and normalized just as DPX-related features were done.

### Classifiers construction

Support vector machine (SVM) is an effective supervised learning model suitable for binary classification [43]. In this study, we used SVM classifiers to distinguish heme binding residues from non-binding residues. These classifiers can be divided into two classes, depending on whether structural information or sequence information was used to build the prediction model. Fifteen structure-based classifiers were constructed using PSSM, RASA, DPX, CX or the combinations of these features. The input of each structure-based classifier is a spatial window of *M *residues containing the target residue and its nearest neighbors obtained by calculating the distances between the *α*-carbons of residues. Alternatively, two sequence-based classifiers were built using amino acid binary pattern and PSSM [27-30]. The input of each sequence-based classifier is a sliding window of *N *consecutive residues centered on the target residue. The optimal values of *M *and *N *were determined by using different widow sizes as input. The LIBSVM package [44] was utilized to implement these SVM classifiers and the radial basis function was selected as kernel. The optimal parameters of each SVM classifier were obtained by combining a grid search with 5-fold cross-validation. By comparing the performances of all the SVM classifiers, we chose the structure-based classifier with the combination of PSSM, RASA, DPX and CX features and the sequence-based classifier with PSSM as the final classifiers.

### Reduction of false positives

Previous studies showed that residues located in ligand binding interfaces are more evolutionarily conserved, and they tend to form spatial clusters [45]. Based on this observation, we developed a post-processing procedure to reduce the number of false positives to further improve the prediction performance. Concretely, for the residues predicted as positives by the structure-based classifier with the combined four features, they were reassigned as negatives if less than *T *(1 ≤ *T *≤ *W*) positive predictions were included in their *W *nearest spatial neighbors. In our experiments, we used different values of *W *and *T *to test the effectiveness of our post-processing procedure. To explain the rationale, we can consider two different scenarios. In both cases, the target residue has been predicted to be a heme binding residue by our structure-based classifier. However, in the first case most of its spatial neighbors are also predicted to be binding residues, but in the second case few of them are predicted to be in the interface. Obviously, the chance that the target residue is indeed a binding residue will be much higher in the first case. No post-processing procedure was applied to the outputs produced by the sequence-based classifier with PSSM, because no remarkable improvement was observed.

### Classifiers combination

**Figure 1:**
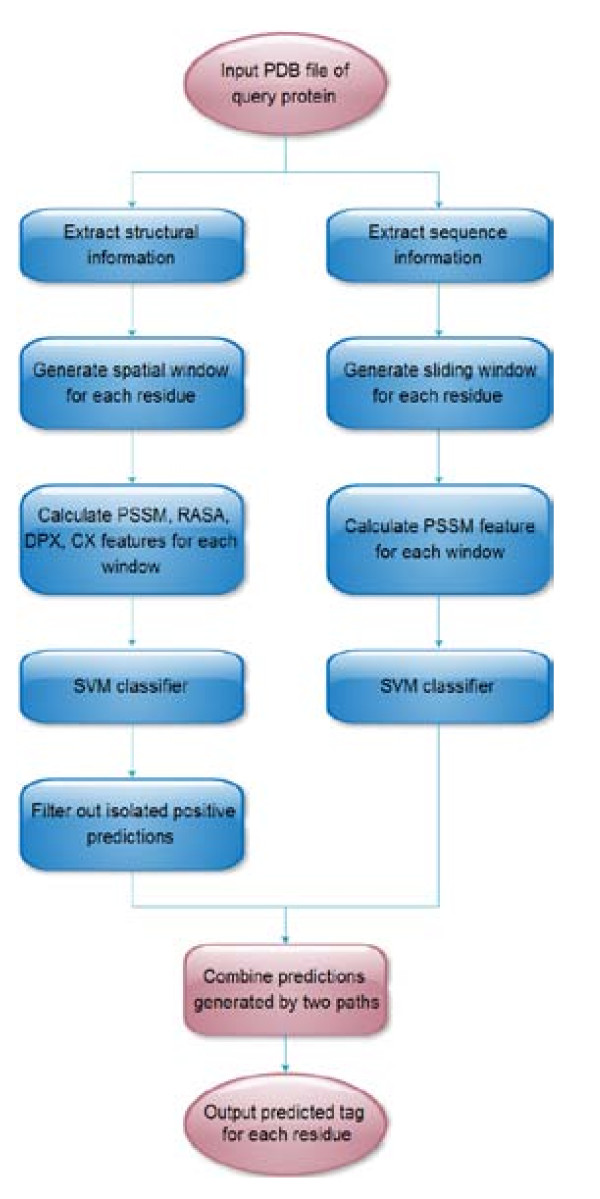
**Schematic diagram of combining outputs generated by individual prediction paths**. The left path contains the structured-based classifier with the combined four features and the post-processing procedure used to reduce false positives, and the right path contains the sequence-based classifier with PSSM.

We proposed a simple voting method to combine the prediction results generated by our final structure-based and sequence-based classifiers in this study. Briefly, a residue was considered as a positive prediction if the filtered output of the structure-based classifier with the combined four features and the output of the sequence-based classifier with PSSM were both labeled as positive; otherwise, it was treated as a negative prediction. Figure 1 shows how to combine the outputs of individual prediction classifiers.

### Training and testing

5-fold cross-validation was conducted on the main dataset and the alternative dataset respectively. In this procedure, the whole dataset were randomly divided into five subsets with an approximately equal number of protein chains. For each run, one subset was left out for testing, while the remaining four subsets were used for training. This process was repeated until all subsets had been tested. The final performance was obtained by averaging the performances of the five subsets. To further assess the robustness of our approach, we used the alternative dataset as a training set to train SVM classifiers which were then used to predict heme binding residues in the independent test set. In our three datasets, the numbers of non-binding residues were much larger than those of binding residues. If all non-binding residues were used for training, the classifiers would be biased to predict a residue as a non-binding residue. Thus, in the process of cross-validation and independent testing, the classifiers were trained using all binding residues and an equal number of non-binding residues extracted randomly from the training set.

### Evaluation measures

In this work, five widely used measures, including recall, precision, accuracy, F1-score and Matthew's correlation coefficient (MCC) were calculated to evaluate the prediction performance of our method. Their definitions are given as follows:(3)(4)(5)(6)(7)

where TP, FP, TN and FN represented true positive (correctly predicted heme binding residue), false positive (non-binding residue incorrectly predicted as binding), true negative (correctly predicted non-binding residue) and false negative (binding residue incorrectly predicted as non-binding), respectively.

## Results and discussion

### Characteristics of heme binding residues

In this study, the proposed prediction algorithm was developed on the basis of the complementary relationship between structural and sequence information. Before using HemeBIND for prediction, we examined the distributions of the following properties of residues located in heme binding interfaces compared with the remainder of the protein, including interface propensity, evolutionary conservation, solvent accessibility, depth, protrusion and spatial clustering of binding residues. In addition, the Kolmogorov-Smirnov test was conducted to evaluate the statistically significant difference. Among the aforementioned attributes, while the depth distributions of heme binding and non-binding residues were most similar, we got a *P*-value of 5.4 × 10^-22^. The *P*-values of the remaining attributes were smaller than that of the depth, indicating that the difference of the distributions was statistically significant for each attribute. The results described herein were derived from the main dataset and similar results were observed when we used the alternative dataset to perform the same analysis (Additional file 2, Figure S1).

**Figure 2:**
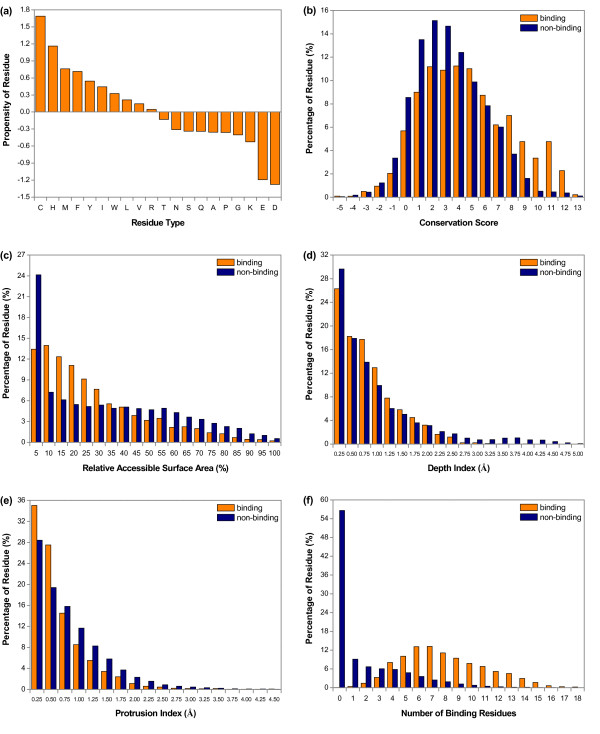
**Characteristics comparison between heme binding and non-binding residues**. (a) Interface propensity, (b) Evolutionary conservation, (c) Solvent accessibility, (d) Depth, (e) Protrusion, (f) Spatial clustering of binding residues. The distributions of (b)-(f) were obtained by dividing all the residues of main dataset into different brackets according to their attribute values and calculating the percentages of binding and non-binding residues in each bracket.

To measure the relative importance of different amino acids in heme binding interfaces, we calculated the interface propensity for each residue type, which is defined as the log ratio between the amino acid frequency in heme binding interface and that in the rest of the protein. From Figure 2(a), it is clear that ten residue types were overrepresented in our dataset, most of which were non-polar or aromatic amino acids. The top four residue types with high propensities were Cys, His, Met and Phe, which is consistent with the results obtained by Smith et al. [46]. Additionally, the other overrepresented residue types (Ile, Val, Trp, Tyr and Arg) reported by their research were also observed in our study.

Previous studies have demonstrated that ligand binding sites are more conserved than non-binding sites during evolution [45]. To check whether heme binding sites have a similar conservation bias, we used the diagonal element of PSSM at each residue position to evaluate the evolutionary conservation of that residue as was done in [47] and calculated the distribution of the conservation scores of the heme binding and non-binding residues. As shown in Figure 2(b), non-binding residues had relatively higher proportions in the -5-4 brackets. However, binding residues dominated the remaining brackets, especially remarkable for the 8-12 brackets. These results suggested that residues involved in heme binding interfaces are more evolutionarily conserved.

Figure 2(c) displays the relative solvent accessibilities of heme binding residues compared with non-binding residues in the main dataset. We found that 78% of heme binding residues had RASAs of less than 40%, while only 64% of non-binding residues were located in the same brackets. When RASA increased over 40%, the percentages of binding residues became smaller than those of non-binding residues. One might expect that binding residues should be more solvent accessible than non-binding residues, but the results showed that this is not the case. Similar observation was reported by Bartlett et al. [48] when they analyzed the solvent accessibilities of catalytic residues in enzyme active sites. The main reason for this phenomenon might be due to the need for correct positioning and restriction of mobility of the residues in these functional sites.

The mean value of all atom DPXs for each residue was calculated and the distribution is given in Figure 2(d). It can be seen that about 26% of heme binding residues lied on the surface of the protein with depths less than 0.25Å, whereas 30% of non-binding residues were observed in this bracket. However, in the 0.5-1.75Å brackets, binding residues appeared more frequently than non-binding residues. Additionally, binding residues rarely had depths greater than 2.5Å, which allows these residues to have some solvent accessibility to interact with the heme molecule whilst remaining mostly buried.

Figure 2(e) shows the distribution of protrusion values. We found that the percentages of binding and non-binding residues with CXs no larger than 0.5Å were 63% and 48%, respectively. But as the protrusion value increased, the proportions of binding residues became smaller than those of non-binding residues. The results indicated that most of heme binding residues have lower CXs compared to non-binding residues. This might be due to the fact that ligand binding residues are usually located in the concavities of a protein.

It has been suggested that evolutionarily conserved residues tend to be clustered in the three-dimensional protein structures [45]. Thus, in a heme protein, it would be expected that the residues involved in heme-protein interactions are conserved and clustered in vicinity of the heme ligands. For each residue, we counted the number of binding residues among its 18 spatially neighboring residues. Figure 2(f) shows that almost 66% of non-binding residues had no more than one binding residue in their 18 neighbors, and the proportion decreased steadily as the number of binding residues increased. Instead, heme binding residues illustrated a completely different distribution. For each binding residue, there was at least one binding residue observed in its neighbors. In the 6-7 brackets, the percentages of binding residues were the highest, indicating that heme binding residues indeed tend to form spatial clusters.

### Determination of optimal window sizes for feature calculation

**Figure 3:**
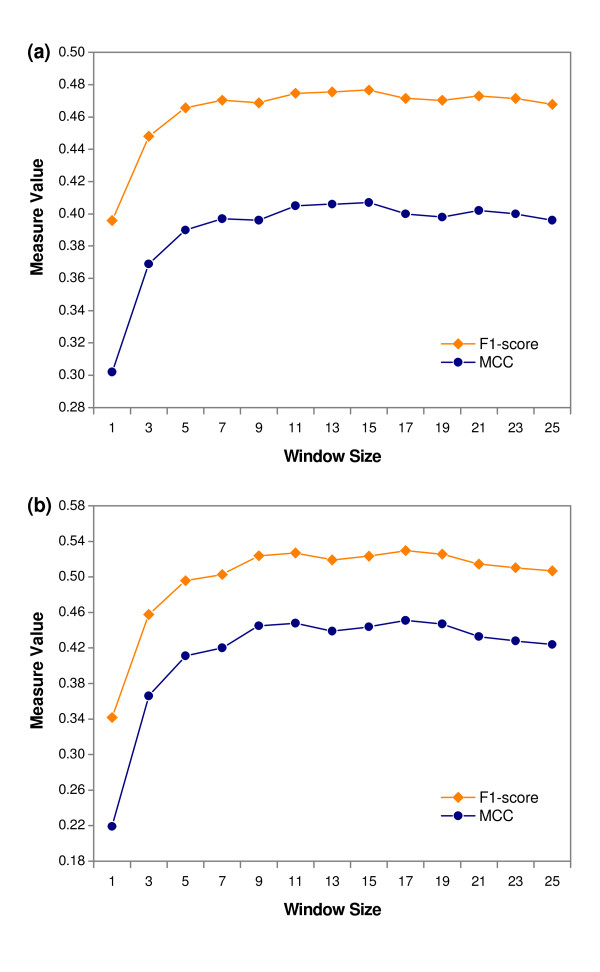
**Performance of using different window size as input**. (a) Structure-based classifier, (b) Sequence-based classifier. The MCC and F1-score were obtained by conducting 5-fold cross-validation on main dataset.

In HemeBIND, the structural context of each residue is reflected by a spatial window of *M *residues. Similarly, the sequence context is reflected by a sliding window of *N *residues. Choosing appropriate window sizes can lead to better prediction performance. In our experiments, the optimal value of *M *for the structure-based classifier with the combined four features (PSSM, RASA, DPX and CX) and that of *N *for the sequence-based classifier with PSSM were determined by testing different window sizes from 1 to 25 on the main dataset. Here MCC and F1-score were used as the main measures to evaluate the performance. As shown in Figure 3, if only the target residue was used as input, the MCC and F1-scores were lower for the two classifiers. However, as we increased the window sizes, the performances of the two classifiers were remarkably improved. This suggested that the local environment around the target residue should be considered when predicting heme binding residues. In addition, we noticed that the best performance was obtained when *M *= 15 for the structure-based classifier. For sequence-based classifier, the prediction performance was peaked when *N *= 17. Therefore, unless otherwise stated, we used *M *= 15 and *N *= 17 as the default window sizes for the structure-based and sequence-based classifiers respectively.

### Performance of structure-based classifiers tested on main dataset

In this study, fifteen structure-based classifiers were constructed for identification of heme binding residues, including four classifiers with a single feature, six classifiers with the combination of two features, four classifiers with the combination of three features and one classifier with the combination of all four features. This allows the comparison of the predictive capabilities of the different features. Moreover, we can discern whether these features are complementary for heme binding residue prediction. The prediction results of 5-fold cross-validation on the main dataset are given in Table 1. It can be seen that PSSM, the sequence feature based on evolutionary conservation, achieved a much better performance compared to structural features, with the MCC of 0.342 and F1-score of 42.64%. Among the three structural features, the CX feature achieved the best performance, the RASA feature was second and the DPX feature gave a relatively inferior performance. But the MCCs and F1-scores of four single-feature based classifiers were all greater than 0.2 and 30% respectively, suggesting that these features can be used to recognize heme binding residues.

**Table 1:** Performance of structure-based classifiers on main dataset

Feature	Recall (%)	Precision (%)	Accuracy (%)	F1-score (%)	MCC
DPX	73.28	22.81	62.97	34.77	0.239
RASA	70.52	28.64	72.36	40.70	0.313
CX	77.25	28.21	70.47	41.29	0.330
PSSM	74.55	29.88	72.98	42.64	0.342
RASA+DPX	76.69	27.82	70.03	40.79	0.323
PSSM+CX	75.66	31.11	74.10	44.04	0.361
PSSM+RASA	75.54	31.24	74.24	44.15	0.362
DPX+CX	80.56	30.40	72.53	44.11	0.370
PSSM+DPX	76.64	33.19	75.98	46.25	0.388
RASA+CX	75.97	33.68	76.64	46.66	0.391
PSSM+RASA+CX	76.11	31.90	74.80	44.89	0.371
RASA+DPX+CX	80.61	32.26	74.62	46.07	0.392
PSSM+RASA+DPX	76.90	33.87	76.54	46.94	0.396
PSSM+DPX+CX	78.81	33.64	76.15	47.10	0.401
PSSM+RASA+DPX+CX	79.08	34.07	76.49	47.56	0.407

More interestingly, we found that combining any two features can improve the prediction performance to a certain degree. Among the six classifiers with the combination of two features, the classifier based on PSSM and DPX and the classifier based on RASA and CX achieved the best performance with the MCC of about 0.39 and F1-score of about 46%. Although the remaining two-feature based classifiers did not perform as well as the two classifiers aforementioned, they were still superior to the classifiers with a single feature. The results implied that these four features contain different and complementary information for heme binding residue prediction.

In addition, we observed that when RASA or CX was incorporated as an additional feature into the classifier based on PSSM and DPX, the MCC and F1-score were slightly raised. However, not all the classifiers with three features obtained a better prediction result. For example, as we added PSSM into the classifier based on RASA and CX, the predictive capability got a little worse. Finally, the classifier with the combined four features achieved the highest MCC of 0.407 and F1-score of 47.56% among all fifteen structure-based classifiers, which confirmed that the complementarity of these four features is beneficial for improving the prediction of heme binding residues.

### Performance of post-processing procedure

After we obtained the predictions generated by the structure-based classifier with the combined four features, a post-processing procedure was used to reduce the false positives in these predictions. Here five values of the number of spatial neighbors (*W *= 6, 10, 14, 18 and 22) were tested. For each value of *W*, we thoroughly tested the value of *T *from 1 to *W *and the optimal value of *T *was determined when the best performance was achieved. From Table 2, it is obvious that compared with the raw predictions, the performances were further optimized by conducting the post-processing procedure with different combinations of *W *and *T *values. Especially for *W *= 18 and *T *= 5, showing the largest increase, the precision, accuracy, F1-score and MCC were improved from 34.07% to 37.14%, 76.49% to 79.24%, 47.56% to 49.76% and 0.407 to 0.427, respectively. Accordingly, in our post-processing procedure, we chose *W *= 18 and *T *= 5 as the default parameters. On the other hand, it is worth mentioning that the post-processing method increased precision at the expense of recall, because some true positives were inevitably reassigned as negatives. However, we think this trade-off is worthwhile, since experimental biologists could pay more attention to the precision measure when they use a classifier to identify heme binding residues in reality.

**Table 2:** Performance of post-processing procedure on main dataset

	Recall (%)	Precision (%)	Accuracy (%)	F1-score (%)	MCC
*W *= 0, *T *= 0^a^	79.08	34.07	76.49	47.56	0.407
*W *= 6, *T *= 1	77.23	35.32	77.77	48.39	0.413
*W *= 10, *T *= 2	77.84	35.74	78.05	48.89	0.420
*W *= 14, *T *= 4	75.75	37.04	79.22	49.62	0.425
*W *= 18, *T *= 5	76.05	37.14	79.24	49.76	0.427
*W *= 22, *T *= 6	76.05	37.09	79.20	49.72	0.426

^a^*W *= 0, *T *= 0 denotes the raw predictions without post-processing.

### Performance of sequence-based classifiers tested on main dataset

Besides the fifteen structure-based classifiers, two sequence-based classifiers were constructed using amino acid binary pattern and PSSM. As shown in Table 3, when amino acid binary pattern was adopted to build the classifier, it achieved a MCC of 0.249 and F1-score of 36.01%. On the other hand, the PSSM feature based classifier obtained a substantial increase in the prediction performance with the MCC of 0.451 and F1-score of 52.97%. Similar performance discrepancy has been observed in the studies of Raghava et al. [27-30], where they utilized these two types of features to identify the binding residues of ATP, GTP, FAD and NAD molecules in protein sequence respectively. The results clearly indicated that when only sequence information is available, evolutionary conservation is very important for the prediction of heme binding residues. Surprisingly, we observed that the sequence-based classifier with PSSM achieved a better performance than the structure-based classifier with the combination of PSSM and three structural features. However, revisiting Figure 3, we found that if the input window only contained the target residue, the structure-based classifier obviously outperformed the sequence-based classifier, indicating that the incorporation of RASA, DPX and CX features indeed provided other useful information for predicting heme binging residues. Thus, the better performance achieved by the sequence-based classifier with increasing window sizes could be attributed to the use of the sliding window. Owing to the fact that heme proteins usually contain some common linear motifs, such as Cys-Xaa-Xaa-Cys-His (CXXCH) in *c*-type heme proteins [49], the sliding window might more effectively reflect the local environment around heme binding residues relative to the spatial window.

**Table 3:** Performance of sequence-based classifiers on main dataset

Feature	Recall (%)	Precision (%)	Accuracy (%)	F1-score (%)	MCC
AA^a^	66.37	24.72	68.30	36.01	0.249
PSSM	63.29	45.64	84.88	52.97	0.451

^a ^AA denotes amino acid binary pattern.

### Performance of the ensemble classifiers

To further improve the performance, the prediction results of the structure-based classifier with the combined four features and those of the sequence-based classifier with PSSM were integrated by a voting method. We compared the combined prediction models with the individual classifiers and the performances are listed in Table 4. It can be seen that a significant increase in prediction performance was achieved by combining the raw predictions of the structure-based classifier and the predictions of the sequence-based classifier (*P*-value < 0.001, Wilcoxon signed-rank test). Compared with the two individual classifiers, the MCC of the combined model were increased by approximate 0.09 and 0.05, respectively. However, we also noted that the combined model obtained a decrease in the recall relative to the individual classifiers, but raised the precision. This was due to the fact that integrating structural and sequence information contributed to the dramatic reduction of false positives, although more true positives converted into false negatives. Additionally, using the filtered predictions of the structure-based classifier to vote, we obtained a slightly better performance with the MCC of 0.51 and F1-score of 57.07%. Therefore, it can be concluded that our combined prediction model for recognizing heme binding residues achieved a satisfactory performance.

**Table 4:** Performance of different prediction models on main dataset

Model^a^	Recall (%)	Precision (%)	Accuracy (%)	F1-score (%)	MCC	FP	TN	FN	TP
STR	79.08	34.07	76.49	47.56	0.407	7824	24888	1061	4018
STR_RFP_	76.05	37.14	79.24	49.76	0.427	6629	26083	1215	3864
SEQ	63.29	45.64	84.88	52.97	0.451	3852	28860	1865	3214
STR+SEQ	55.87	57.48	88.44	56.55	0.500	2128	30584	2241	2838
STR_RFP_+SEQ	54.08	60.74	89.03	57.07	0.510	1814	30898	2332	2747

^a^STR, RFP and SEQ denote structure-based classifier, reducing false positives and sequence-based classifier respectively.

### Performance of our classifiers tested on alternative dataset

To test whether our classifiers can effectively identify heme binding residues in another dataset, we conducted 5-fold cross-validation on the 75 heme proteins collected by Fufezan et al. [2] and the results are given in Table S1 (Additional file 2). As shown in this table, the ranking of the predictive capabilities of the different classifiers was almost consistent with that achieved on the main dataset, with exception of the structure-based classifiers with two features. Additionally, as expected, the performance of each classifier was not as good as that of the corresponding classifier tested on the main dataset, which could be due to the relatively small number of samples in the training set. Even so, when the combined prediction model was used to predict heme binding residues in the alternative dataset, we obtained a reasonable performance with the MCC of 0.465 and F1-score of 52.94%. The results demonstrated that our method performed well on different datasets.

### Independent testing

We trained our classifiers using the alternative dataset and used them to recognize heme binding residues of the newly added non-homologous heme proteins during the last three years. Furthermore, since the heme proteins can interact with either a single heme ligand or multiple heme ligands, we attempted to test whether our approach can be used to predict the binding residues in these two types of heme proteins. Table 5 shows the results of different classifiers tested on the independent test set which is composed of 62 single-heme proteins and 10 multi-heme proteins. It can be observed that compared with the individual classifiers, the combined prediction model still achieved a better performance not only for the single-heme proteins, but also for the multi-heme proteins. Interestingly, focusing on the multi-heme proteins, we can find that the performance of the structure-based classifier was no worse than that of the sequence-based classifier, and was much better than that of the corresponding classifier tested on single-heme proteins. The possible reason is that together with the spatial window, the structural attributes, such as solvent accessibility, depth and protrusion, can more adequately reflect the geometric environment of binding residues in the multi-heme proteins. In addition, for the whole independent test set, the experimental results were in agreement with those of 5-fold cross-validation on the main dataset and the alternative dataset, and the final prediction model achieved a MCC of 0.504 and F1-score of 56.87%. In summary, our prediction model is robust and promising for the prediction of heme binding residues.

**Table 5:** Performance of different prediction models on independent test set

Subset	Model^a^	Recall (%)	Precision (%)	Accuracy (%)	F1-score (%)	MCC
Single-heme	STR	76.05	28.72	77.06	41.69	0.366
	STR_RFP_	72.10	31.44	80.03	43.78	0.382
	SEQ	58.86	39.35	85.78	47.17	0.404
	STR+SEQ	52.28	52.75	89.80	52.51	0.468
	STR_RFP_+SEQ	50.54	55.78	90.34	53.03	0.477
Multi-heme	STR	87.07	51.48	69.66	64.70	0.454
	STR_RFP_	86.56	52.60	70.80	65.43	0.466
	SEQ	63.14	59.85	74.70	61.45	0.427
	STR+SEQ	58.55	69.53	78.57	63.57	0.489
	STR_RFP_+SEQ	58.25	70.18	78.76	63.66	0.493
All	STR	80.13	34.93	75.83	48.65	0.412
	STR_RFP_	77.45	37.72	78.50	50.73	0.431
	SEQ	60.44	45.36	83.94	51.83	0.431
	STR+SEQ	54.60	58.34	87.94	56.41	0.495
	STR_RFP_+SEQ	53.39	60.82	88.42	56.87	0.504

^a^STR, RFP and SEQ denote structure-based classifier, reducing false positives and sequence-based classifier respectively.

### Comparison with other methods

In this section, we compared HemeBIND with Ligsite^CSC ^[17] and ConCavity [19], which are both geometry-based prediction methods and incorporate residue evolutionary conservation to improve performance. We downloaded the prediction results of 59 heme proteins used in our independent testing from the web servers of these two methods. The performance comparison of different methods is summarized in Table 6. We can see that Ligsite^CSC ^performed quite poorly compared with HemeBIND and ConCavity, which is consistent with the results reported by Capra et al. [19]. They illustrated that the existing structure-based servers (including Ligsite^CSC^) that focus on pocket detection do not outperform a simple sequence conservation approach in finding ligand binding residues. In ConCavity algorithm, Jensen-Shannon divergence (JSD) is used to score the evolutionary conservation of each residue and the residues with a higher JSD score are considered as potential ligand binding residues. From Table 6, it is obvious that our sequence-based method was superior to JSD method, which is possibly due to the fact that we take into consideration the sequence context of target residues used to capture the motif information in heme proteins. As ConCavity directly integrated conservation into the search for pockets, the performance was better than that of HemeBIND. However, from a methodological perspective, HemeBIND is constructed on the basis of machine learning algorithm, which is different from ConCavity. Therefore, our method and ConCavity can complement each other for heme binding residue prediction.

**Table 6:** Performance comparison of different prediction methods

	Ligsite^CSC^		ConCavity		HemeBIND	
	SEQ^a^	STR+SEQ^b^	SEQ	STR+SEQ	SEQ	STR+SEQ
Recall (%)	N/A	12.59	31.66	65.26	61.64	54.71
Precision (%)	N/A	34.88	30.63	61.55	46.12	61.35
Accuracy (%)	N/A	83.31	78.93	88.64	83.39	88.00
F1-score (%)	N/A	18.50	31.14	63.35	52.76	57.84
MCC	N/A	0.133	0.187	0.567	0.436	0.510

^a^SEQ denotes that the prediction method uses sequence information alone.

^b^STR+SEQ denotes that the prediction method uses both structural and sequence information.

### Case studies

**Figure 4:**
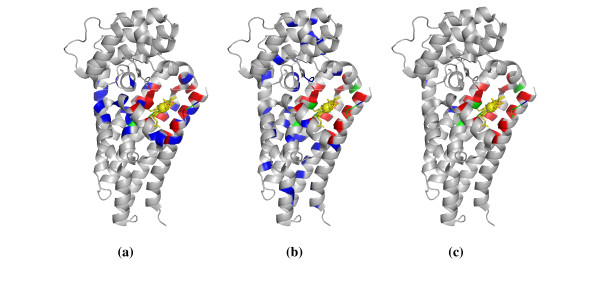
**Visualization of prediction results for chain A of protein complex 2V7I**. (a) Structure-based model, (b) Sequence-based model, (c) Combined model. The following color scheme is used: heme in yellow, true positives in red, false positives in blue, false negatives in green.

To further demonstrate the effectiveness of our approach, we selected two heme proteins from the independent test set to visualize the prediction results using the PyMOL package [50]. The first example is the enzyme PrnB (2V7I:A), which plays a crucial role in the pyrrolnitrin biosynthesis pathway [51]. The structure of PrnB established that it is a member of the *b*-type heme dependent dioxygenase superfamily. The prediction results of this enzyme generated by structure-based, sequence-based and combined prediction models are provided in Figure 4. It is clear that the structure-based and sequence-based prediction models had correctly identified most of the residues interacting with the heme ligand, but meanwhile they got 26 and 37 false positives respectively. However, by combining structural and sequence information, we only obtained 3 false positives, although the number of true positives was slightly decreased. Overall, our combined prediction model achieved an excellent performance with the recall of 70.00%, precision of 87.50%, accuracy of 96.54%, F1-score of 77.78% and MCC of 0.765. These results further demonstrated that the individual models provide largely complementary information, which can be combined to improve prediction performance.

**Figure 5:**
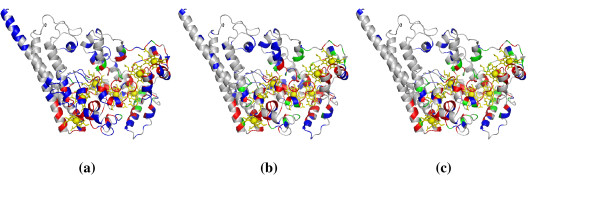
**Visualization of prediction results for chain A of protein complex 1FGJ**. (a) Structure-based model, (b) Sequence-based model, (c) Combined model. The color scheme is the same as that of Figure 4.

Besides the enzyme PrnB, the hydroxylamine oxidoreductase (1FGJ:A) that interacts with seven *c*-type heme ligands was chosen as another example [52]. This enzyme, which converts hydroxylamine molecule into a nitrite, is a key component in respiratory chain. As given in Figure 5, when the combined prediction model was used, the number of false positive predictions was drastically reduced and we obtained an acceptable result for this heme protein with the recall of 65.91%, precision of 63.04%, accuracy of 80.76%, F1-score of 64.44% and MCC of 0.513. Therefore, the proposed HemeBIND algorithm can be used to identify the binding residues of both single-heme and multi-heme proteins.

## Conclusions

In this study, we proposed HemeBIND, the first specialized algorithm for heme binding residue prediction, by combining structural and sequence information. Through systematic analysis of heme binding interfaces, we found that several sequence and structural attributes, such as evolutionary conservation, solvent accessibility, depth and protrusion can distinctly reflect the differences between heme binding regions and the rest of the protein. Based on this finding, the attributes mentioned above were separately used or combined to construct structure-based and sequence-based classifiers to identify the residues located in binding regions. Experimental results showed that evolutionary conservation is an indispensable factor for predicting heme binding residues, but not sufficient by itself, especially when structural information is available. Integrating structural attributes with evolutionary conservation yielded a remarkable improvement in performance over conservation alone. In summary, our study not only presents a new method to recognize heme binding residues, but also provides valuable insights into specific ligand binding site prediction.

## Competing interests

The authors declare that they have no competing interests.

## Authors' contributions

RL designed the study, implemented the algorithm, performed the analysis and drafted the manuscript. JJH designed the study and drafted the manuscript. Both authors read and approved the final manuscript.

## Supplementary Material

Additional file 1 **Datasets used in this study**. The heme proteins used in the three datasets are listed in Table S1-S3, respectively. Click here for file

Additional file 2 **Analysis and performance of alternative dataset**. The characteristic analysis of alternative dataset is given in Figure S1. The performances of different prediction models are given in Table S1. Click here for file
