# Supplementary material for: HemeBIND: a novel method for heme binding residue prediction by combining structural and sequence information
Source: BMC Bioinformatics. 2011 May 26;12:207. doi: 10.1186/1471-2105-12-207 (PMC3124436; doi:10.1186/1471-2105-12-207)
Supplement: Additional file 1 — Datasets used in this study. The heme proteins used in the three datasets are listed in Table S1-S3, respectively. [file 1471-2105-12-207-S1.PDF]

Table S1 The list of 141 chains used in main dataset

1AOF:A	1ETP:A	1LM3:B	1TU9:A	2BVJ:A	2NWB:A	2VR0:A	3DBG:A
1B7V:A	1EYS:C	1MN1:A	1U17:A	2C8S:A	2O09:A	2VRY:A	3DP5:A
1BBH:A	1FCB:A	1MZ4:A	1U5U:A	2CZS:A	2O6P:A	2VXH:A	3E4W:A
1BVB:A	1FCD:C	1N5U:A	1UX8:A	2D0T:A	2OYY:A	2VYW:A	3EH4:A
1CCA:A	1FGJ:A	1N97:A	1V9Y:A	2D3Q:A	2PBJ:A	2W31:A	3EMM:A
1CF9:A	1FOC:A	1NEK:C	1VF5:C	2DGE:A	2Q7A:A	2W3D:A	3EQM:A
1CPO:A	1FS9:A	1NTF:A	1W2L:A	2E84:A	2Q8P:A	2WIV:A	3FVB:A
1CQX:A	1FT9:A	1O1M:A	1W4W:A	2EFB:A	2Q9F:A	2WTG:A	3GAS:A
1CYO:A	1H1O:A	1OR4:A	1X3K:A	2FW5:A	2QBL:A	2WV2:A	3H33:A
1D0C:A	1HBG:A	1PXX:A	1X7U:A	2G5G:X	2QD2:A	2Z6F:A	3H8T:A
1D4C:A	1ITH:A	1Q16:C	1X9F:C	2HI4:A	2QFN:A	2ZON:G	3HF2:A
1D7C:A	1IZO:A	1QHU:A	1XBN:A	2HQ2:A	2QJY:A	2ZOO:A	3HQ6:A
1D8U:A	1J77:A	1RWJ:A	1YIQ:A	2IG3:A	2QJY:B	351C:A	3HX9:A
1DII:C	1JBQ:A	1SCT:B	1YQ3:C	2IIZ:A	2R79:A	3A15:A	3K9V:A
1DLY:A	1JJU:A	1SOX:A	1Z9N:A	2ITF:A	2RCH:A	3B42:A	3LGM:A
1DP6:A	1JNI:A	1SP3:A	2A3M:A	2IVF:C	2V7I:A	3B6H:A	
1DVE:A	1KQF:C	1T2B:A	2BK9:A	2J7A:C	2VE3:A	3BZ1:E	
1DW0:A	1KR7:A	1TQN:A	2BS2:C	2NW7:A	2VEB:A	3CQV:A	

Table S2 The list of 75 chains used in alternative dataset

1A6M:A	1GWE:A	1JFB:A	1PBY:A	1TU9:A	1YRC:A	2FKZ:A	2IVF:C
1ASH:A	1GWU:A	1JNI:A	1PL3:A	1U55:A	256B:A	2FWT:A	2NW8:A
1C75:A	1H97:A	1KQF:C	1PO5:A	1U5U:A	2BK9:A	2G5G:X	2NWB:A
1D0C:A	1IRD:B	1M1Q:A	1PPJ:C	1V07:A	2BKM:A	2GDM:A	2VHB:A
1D2V:C	1IT2:A	1MJ4:A	1Q1F:A	1V9Y:A	2BOQ:A	2H88:C	3PGH:A
1DK0:A	1IW0:A	1N40:A	1QHU:A	1X8Q:A	2BS2:C	2H88:D	
1DLW:A	1IZO:A	1N5U:A	1RWJ:A	1X8V:A	2CIW:A	2HQ2:A	
1EW6:A	1J0P:A	1N97:A	1SCT:A	1XJ4:A	2CZS:A	2IIZ:A	
1FS7:A	1J77:A	1OR4:A	1SI6:X	1XME:A	2D0T:A	2IJ2:A	
1FT5:A	1JF4:A	1OS6:A	1SP3:A	1Y5I:C	2EUT:A	2ITF:A	

Table S3 The list of 72 chains used in independent test set

1AOF:A	1FGJ:A	1T2B:A	2C8S:A	2PBJ:A	2VE3:A	2ZOO:A	3E4W:A
1BBH:A	1FOC:A	1TQN:A	2D3Q:A	2Q7A:A	2VEB:A	351C:A	3EMM:A
1CYO:A	1FT9:A	1VF5:C	2DGE:A	2Q8P:A	2VR0:A	3A15:A	3EQM:A
1DII:C	1H1O:A	1W2L:A	2E84:A	2Q9F:A	2VXH:A	3B42:A	3GAS:A
1DW0:A	1ITH:A	1X3K:A	2IG3:A	2QD2:A	2W31:A	3B6H:A	3H8T:A
1ETP:A	1JBQ:A	1X7U:A	2J7A:C	2QJY:B	2W3D:A	3BZ1:E	3HQ6:A
1EYS:C	1MZ4:A	1X9F:C	2O09:A	2R79:A	2WIV:A	3CQV:A	3HX9:A
1FCB:A	1NEK:C	1YIQ:A	2O6P:A	2RCH:A	2Z6F:A	3DBG:A	3K9V:A
1FCD:C	1SCT:B	1Z9N:A	2OYY:A	2V7I:A	2ZON:G	3DP5:A	3LGM:A
