# Supplementary material for: HemeBIND: a novel method for heme binding residue prediction by combining structural and sequence information
Source: BMC Bioinformatics. 2011 May 26;12:207. doi: 10.1186/1471-2105-12-207 (PMC3124436; doi:10.1186/1471-2105-12-207)
Supplement: Additional file 2 — Analysis and performance of alternative dataset. The characteristic analysis of alternative dataset is given in Figure S1. The performances of different prediction models are given in Table S1. [file 1471-2105-12-207-S2.PDF]

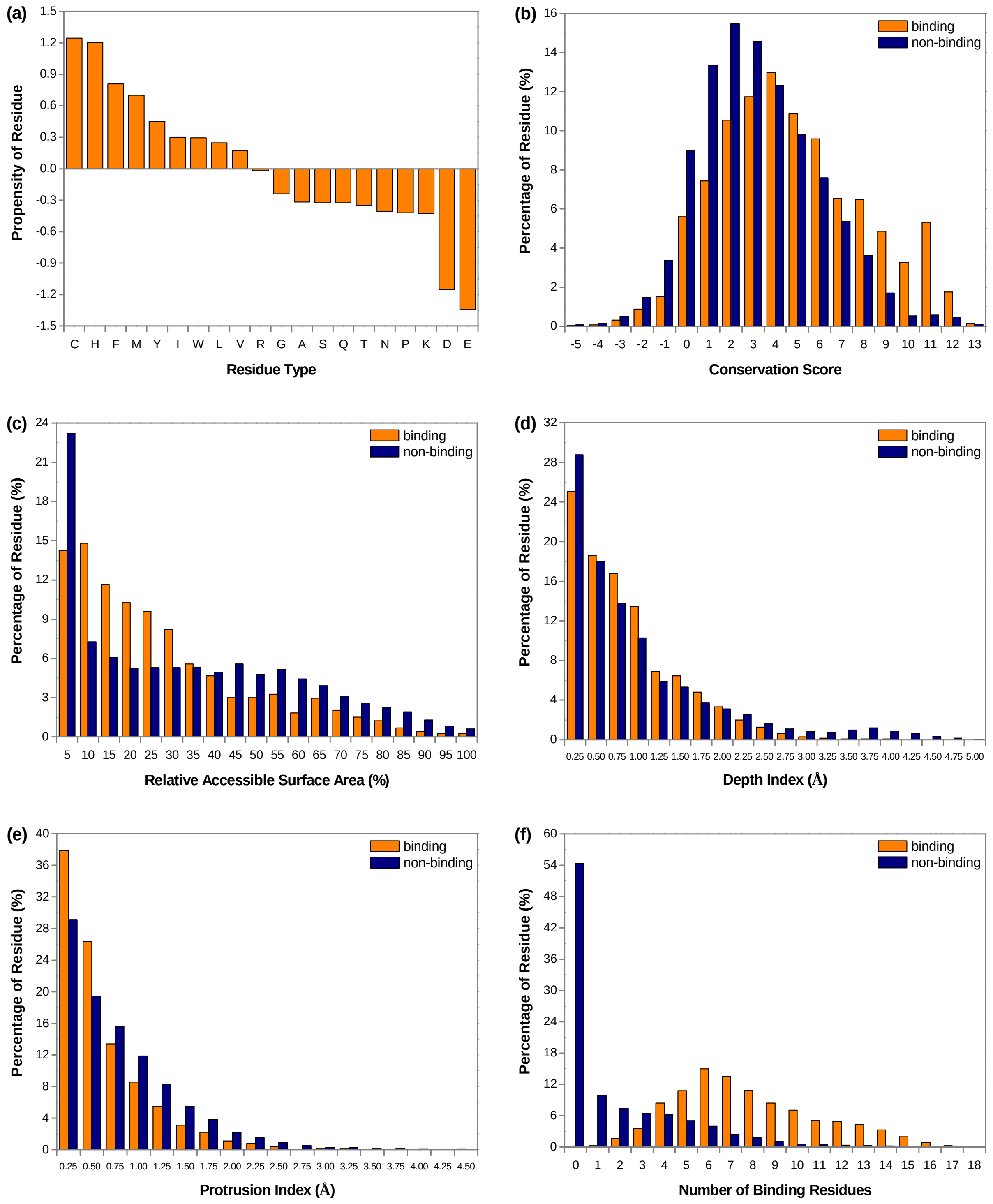

Figure S1 Characteristics comparison between heme binding and non-binding residues of alternative dataset. (a) Interface propensity, (b) Evolutionary conservation, (c) Solvent accessibility, (d) Depth, (e) Protrusion, (f) Spatial clustering of binding residues.

Table S1 - Performance of different prediction models on alternative dataset

Model[a] (Feature)	Recall (%)	Precision (%)	Accuracy (%)	F1-score (%)	MCC
DPX	72.50	23.00	63.17	34.83	0.238
RASA	70.97	25.97	68.63	37.98	0.278
CX	77.19	26.10	66.99	38.90	0.299
PSSM	72.81	28.01	69.88	40.14	0.306
RASA+DPX	74.02	27.77	70.33	40.32	0.312
PSSM+RASA	73.14	29.17	70.90	41.23	0.320
PSSM+CX	74.84	28.67	70.37	41.17	0.322
RASA+CX	76.14	29.60	71.73	42.44	0.341
DPX+CX	78.91	29.10	70.77	42.41	0.345
PSSM+DPX	75.61	30.62	72.35	43.17	0.348
PSSM+RASA+CX	75.13	29.32	70.94	41.82	0.330
RASA+DPX+CX	78.12	29.87	71.92	43.12	0.352
PSSM+RASA+DPX	76.28	31.28	72.91	43.92	0.358
PSSM+DPX+CX	76.17	31.75	73.73	44.46	0.365
PSSM+RASA+DPX+CX (STR)	77.27	31.94	73.82	44.90	0.371
STR_{RFP}	74.41	34.44	76.45	46.66	0.388
SEQ	61.17	41.52	82.53	48.89	0.404
STR+SEQ	53.73	53.49	86.85	52.68	0.457
STR_{RFP}+SEQ	52.21	56.02	87.37	52.94	0.465

[a]STR, RFP and SEQ denote structure-based classifier, reducing false positives and sequence-based classifier respectively.
